# Interpreting the Intensity of Vocal Emotions Across Cultures

**DOI:** 10.1111/sjop.70081

**Published:** 2026-02-19

**Authors:** Yachan Liang, Roeland van Hout, Vincent J. van Heuven

**Affiliations:** ^1^ Centre for Linguistics Leiden University Leiden the Netherlands; ^2^ Centre for Language Studies Radboud University Leiden the Netherlands

**Keywords:** arousal, basicness, cross‐cultural, Dutch, in‐group bias, intensity, Korean, valence, vocal emotions

## Abstract

Intensity is a fundamental dimension of emotions that affects their perception. However, theoretical and empirical studies on intensity, particularly in the vocal domain, remain limited. Furthermore, research on the effects of emotional dimensions (e.g., arousal, valence, and basicness) on intensity ratings remains sparse. This study investigates cross‐cultural intensity ratings using the Demo (Dutch) and Koremo (Korean) corpora, each based on eight actors vocalizing eight emotions by pronouncing a pseudo‐sentence. Both corpora were rated on intensity by Dutch and Korean listeners. The first goal is to examine the presence of in‐group bias in intensity ratings across all responses and correct responses. The second goal is to assess the relative contributions of arousal, valence, and basicness to intensity ratings across all responses and correct responses. To achieve these goals, we conducted an intensity‐rating experiment in which all participants rated the intensity of each emotion they perceived on a 4‐point scale (1 = low intensity; 4 = high intensity). Participants consisted of 31 native Dutch listeners and 24 native Korean listeners, none of whom had prior knowledge of the other group's culture or language. Our results corroborate earlier findings and shed new light on intensity ratings of vocal emotions. First, contrary to previous findings, we did not find an in‐group bias in intensity ratings, such that neither listener group gave higher ratings to emotions produced in their native language than in the unknown language. Second, intensity ratings were higher for high‐arousal than for low‐arousal emotions, for negative than for positive emotions, and for basic than for non‐basic emotions. Notably, intensity ratings are more strongly correlated with arousal and basicness than valence, supporting earlier findings that high‐arousal emotions are characterized by increased intensity. Despite the significant effects of arousal, valence, and basicness on intensity ratings, these dimensions do not yield a successful dichotomy of emotions in terms of intensity, since some emotions violate the general patterns of intensity ratings based on these three dimensions. Additionally, intensity ratings were higher for correct than incorrect responses. Together, these findings contribute to a better understanding of the role of intensity in vocal emotion across cultures. Instead of an in‐group bias, listeners tended to rate specific emotions as more intense, even when expressed in a non‐native language. Although intensity ratings were affected by arousal, valence, and basicness, none of these dimensions provided a strict dichotomy of the eight emotions in intensity, since intensity ratings for certain emotions cannot be reliably predicted from the general patterns. Together, these findings contribute to a better understanding of the role of intensity in vocal emotion across cultures.

## Key Points

- Listeners do not consistently rate emotions produced in their own language as more intense than those produced in the unknown language in a cross‐cultural setting.
- Intensity ratings vary more systematically with arousal and basicness rather than valence.
- Binary emotional dimensions fail to fully account for variation in intensity ratings across emotions.

## 1 Introduction

Emotions are ubiquitous in daily life and play a crucial role in interpersonal communication (Hall et al. 2009; Trampe et al. 2015). The fundamental role of emotions in communication has promoted exploration into the complexity of emotions (Elfenbein and Ambady 2003; Larsen et al. 1987; Liu et al. 2012). Most studies have mainly investigated emotions from a discrete approach (Biehl et al. 1997; Ekman and Friesen 1969; Ekman et al. 1987; Elfenbein 2013; Elfenbein et al. 2002; Elfenbein and Ambady 2002; Laukka et al. 2013; see Laukka and Elfenbein 2021 for a review), although some have explored emotions from a dimensional approach (Barrett 1998; Mozziconacci 2002; Russell 1980). Emotions not only differ in quality (intended category) but also in quantity (intensity/strength), ranging from subdued to intense (Flett et al. 1986; Larsen and Diener 1987; Laukka et al. 2005). For instance, anger can vary from mild irritation to intense rage (Spielberger et al. 1995). Misinterpretations of emotional intensity may lead to misunderstandings and even conflicts (Guerrero and La Valley 2006). Therefore, intensity is classified as an important dimension of emotions (Larsen and Diener 1985). Despite these findings, knowledge on intensity remains limited, especially in the vocal domain (Frijda et al. 1992; Reisenzein 1994). The present study, therefore, examines the perception of intensity from a dimensional approach, aiming to provide a more comprehensive understanding of the role of intensity in vocal emotions, especially in a cross‐cultural setting.

### 1.1 The Intensity of Emotions

Emotions are never produced neutrally. Instead, they are always expressed with some intensity (Mesquita and Frijda 1992; Sonnemans and Frijda 1994). Intensity refers to the strength or magnitude of individuals' responses evoked by emotions (Bänziger and Scherer 2005; Diener et al. 1985; Larsen and Diener 1987; Sonnemans and Frijda 1994). Intensity measures how strongly the receiver perceives an emotion. People react to emotions with stronger intensity sooner than to those with weaker intensity (Kommattam et al. 2019). The intensity of emotion affects individuals' physiological and behavioral responses, such as decision‐making (Frijda et al. 1992; Sonnemans and Frijda 1994). For example, highly intense emotions can trigger increased heart rate and blood pressure in the receiver.

### 1.2 Cross‐Cultural Perception of Emotional Intensity

Research on cross‐cultural perception of emotional intensity has predominantly concentrated on the visual domain (e.g., facial expressions), (Holz et al. 2021; Juslin and Laukka 2001; Morningstar et al. 2021; Zhang and Pell 2022). Findings reveal that both universal and culture‐specific factors affect intensity ratings (Ekman et al. 1987; Kommattam et al. 2019; Matsumoto and Ekman 1989). These studies can be broadly divided into two categories; that is, (1) objective recognition accuracy based on stimulus intensity, and (2) subjective judgment of intensity.

#### 1.2.1 Objective Recognition Accuracy Based on Intensity

Recognition accuracy of emotions increases with stimulus intensity, but this pattern varies across emotions (Hess et al. 1997; Juslin and Laukka 2001; Shioiri et al. 1999). In the visual domain, Hess et al. (1997) found that while recognition accuracy of most basic facial expressions improved linearly with physical intensity, happiness was accurately identified at a lower intensity level. Moreover, Shioiri et al. (1999) reported that Japanese participants gave higher intensity ratings to facial expressions than Americans but had lower recognition accuracy.

In the vocal domain, Juslin and Laukka (2001) discovered that listeners can easily decode emotions with stronger intensity than those with weaker intensity. However, this study has focused only on two levels of intensity (strong and weak). Expanding on this, Morningstar et al. (2021) manipulated the intensity of vocal emotions in 10% increments, ranging from 0% (neutral) to 100% (full‐intensity). Recognition accuracy of some emotions (e.g., anger) increased linearly with intensity, whereas recognition accuracy of happiness remained stable at low levels but increased at high levels.

However, Holz et al. (2021) argued that intensity plays a paradoxical role in emotion recognition. While emotions of moderate and strong intensity are accurately identified, those of peak intensity become ambiguous and difficult to recognize. One possible reason is that emotions with peak intensity may involve more than one emotion, making it difficult to classify them as a single emotion.

#### 1.2.2 Subjective Intensity Judgments Based on Stimulus Intensity

Cultural and linguistic factors affect how individuals rate emotional intensity in both visual and vocal domains, displaying different evidence for an in‐group bias in a cross‐cultural setting. Ekman et al. (1987) found that Western participants gave higher intensity ratings to basic emotions than non‐Western participants for Caucasian facial expressions. However, Matsumoto and Ekman (1989) noticed inconsistent in‐group bias across emotions: American participants consistently gave higher intensity ratings than Japanese participants to each emotion except disgust, whereas Japanese participants consistently rated disgust as the most intense emotion. More recently, in a meta‐analysis, Kommattam et al. (2019) found that participants rated emotions produced by in‐group members as more intense than those produced by out‐group members. Notably, some facial expressions that are difficult to recognize, that is, contempt, embarrassment, and pride, were rated as less intense when evaluated by out‐group members. These differences were attributed to cultural influence, supporting the idea that cultural norms affect emotion recognition. Therefore, Kommattam et al. (2019) proposed the notion of *in‐group bias*, indicating that individuals rate emotions produced within their culture as more intense than those produced in an unfamiliar culture. Zhang and Pell (2022) adopted a fully cross‐cultural design and found an overall in‐group bias for both Canadian and Mandarin listeners. Particularly, both listener groups gave higher intensity ratings to anger and fear than to happiness and sadness. However, no in‐group intensity bias was found for sadness (Canadian listeners) and for both anger and fear (Mandarin listeners).

Taken together, previous studies on intensity ratings of facial expressions present different views on the in‐group bias of intensity ratings. Ekman et al. (1987) and Kommattam et al. (2019) reported higher intensity ratings of facial emotions for in‐group than for out‐group members. Notably, both studies provided participants with the corresponding emotion labels for each facial expression, informing participants of the specific emotion they were rating. However, Matsumoto and Ekman (1989) found no evidence for the in‐group bias in intensity ratings in two separate experiments—one with and one without showing the labels of the intended emotions.

### 1.3 The Relationship Between Intensity and Emotional Dimensions

In addition to investigating intensity separately, studies have examined intensity with other emotional dimensions, as emotions are intricate psychological experiences with multiple dimensions (Barrett and Russell 2014; Russell 1980, 2009; Russell and Barrett 1999). One of the most well‐known models is the circumplex model, which classifies emotions into two fundamental dimensions: arousal and valence (Russell 1980). Arousal, also referred to as activation, is the perceivers' physiological responses caused by emotions, ranging from low‐arousal to high‐arousal (Russell and Barrett 1999). On the other hand, valence is the perceivers' personal experience affected by emotions, which can be either positive (pleasant) or negative (unpleasant). Furthermore, other theories propose that intensity should be added as one of the emotional dimensions (Larsen and Diener 1987; Smith and Ellsworth 1985). Intensity is the strength or magnitude of the emotional experience (Brehm 1999), which is arguably related to arousal (Laukka et al. 2005; Mesquita and Frijda 1992; Reisenzein 1994). Although intensity and arousal are closely related, they are not interchangeable. While intensity concentrates on the overall strength of emotion, arousal emphasizes the physiological activation of perceivers elicited by emotion (Sonnemans and Frijda 1995; Zsidó 2024). For instance, intense emotions can be either high‐arousal or low‐arousal. Likewise, positive and negative emotions can be either intense or mild.

Basicness is another important attribute of emotions, although there is debate over whether it should be considered one of the dimensions of emotions. As mentioned above, emotion theory distinguishes six basic emotions (anger, disgust, fear, happiness, sadness, and surprise), which are universally recognized. Basic emotions are universal in the sense that they are expressed and understood in all cultures worldwide (Ekman 1992).

Extending this work, Laukka et al. (2005) investigated vocal emotions from a dimensional approach and found that intensity is positively related to arousal (activation) in terms of ratings by listeners. However, their study employed a “many‐to‐one” design rather than a fully cross‐cultural design, as they presented vocal emotions produced by two groups of speakers (British English and Swedish) to only Swedish listeners.

Collectively, then, arousal, valence, intensity, and basicness categorize emotions along different dimensions, providing a multidimensional framework for better understanding the complexity of emotions. However, compared to these dimensions, intensity has been less studied, and our understanding of the interplay between intensity, arousal, valence, and basicness remains limited. To address this problem, it is necessary to explore the perception of emotional intensity and the extent to which it correlates with arousal, valence, and basicness.

### 1.4 The Present Study

This study investigates intensity ratings in Dutch and Korean listeners, whose cultures and languages are typologically different. It has two primary goals. The first goal aims to examine the in‐group bias in intensity ratings. According to the notion of intensity bias, individuals tend to give higher intensity ratings to emotions expressed by members of their own group than to those expressed by out‐group members (Ekman et al. 1987; Kommattam et al. 2019). Therefore, we hypothesize that both listener groups will exhibit an in‐group bias in intensity ratings. First, we test the in‐group bias across all responses, including correct and incorrect responses. We hypothesize that both listener groups will give higher intensity ratings to emotions expressed in their native language than to those expressed in the unknown language (Hypothesis 1). Second, we test the in‐group bias across correct responses. Previous research on intensity ratings presented participants with corresponding emotion labels, which may have affected their ratings. However, in our materials, listeners were asked to rate the intensity of emotions without knowing the specific emotion (Goudbeek and Broersma 2010). To avoid the effect of emotion labels on intensity ratings, we further examine intensity ratings on correct responses only. Based on the literature reviewed above, we hypothesize that there will exist an in‐group bias across correct responses (Hypothesis 2).

The second goal aims to examine the effect of arousal, valence, and basicness on intensity ratings across all responses and correct responses. First, we examine the effect of Arousal on intensity ratings. According to the literature reviewed above, arousal is positively related to intensity (Laukka et al. 2005). Therefore, we hypothesize that intensity ratings will be higher for high‐arousal than low‐arousal emotions (Hypothesis 3). Second, we examine the effect of Valence on intensity ratings. Negative emotions are more closely associated with higher intensity than positive emotions (Schröder et al. 2001), and emotions with higher intensity are typically recognized as negative rather than positive (Scherer 2003). Therefore, we hypothesize that intensity ratings will be higher for negative than positive emotions (Hypothesis 4). Third, we examine the effect of Basicness on intensity ratings. According to the emotion theory, there is a small number of emotions shared by all humans, which cause fixed behavioral responses (Ekman 1972, 1992; Ekman et al. 1969). Basic emotions include more negative (anger, fear, sadness, and disgust) than positive emotions (happiness/joy), while surprise can be either positive or negative (Ekman 1992). In this study, we included four basic emotions, one positive (i.e., joy), and the other three (anger, fear, and sadness) negative. Since negative emotions are usually rated as more intense than positive emotions (see above, see also Kuppens et al. 2008), we hypothesize that intensity ratings will be higher for basic than non‐basic emotions (Hypothesis 5).

## 2 Method

To address the above research questions, we conducted an intensity rating experiment. During the experiment, all participants were asked to rate the intensity of each emotion they perceived on a four‐point scale from 1 (low intensity) to 4 (high intensity).

### 2.1 Participants

Two groups of participants took part in this experiment. Thirty‐one native listeners of Dutch (27 females, 4 males, age: *M* = 20.87, SD = 2.17) were students at Radboud University, the Netherlands; and 24 native listeners of Korean (12 females, 12 males, age: *M* = 23.46, SD = 2.59) were students at the University of Seoul, Korea. Before the experiment, each participant filled in a questionnaire to confirm they had no prior knowledge of the other group's culture or language. None of them reported any hearing problems. All participants got a small payment or course credits as a reward for their participation.

### 2.2 Stimuli

The auditory materials were vocal expressions from the Demo and Koremo (Dutch emotions and Korean emotions) corpus (Broersma et al. 2025). Each language corpus is based on eight emotions produced by eight actors (64 stimulus types). Each stimulus type was recorded twice (giving two tokens), resulting in a total of 128 portrayals per language (Table 1). For more information regarding the corpora and the recording procedure, see Liang et al. (2025). All emotions were portrayed on the pseudo‐sentence “/nuto hɔm sɛpikɑŋ/”, which is phonologically compatible in Dutch and Korean.^1^ Using pseudo‐sentences avoids semantic cues that may affect intensity ratings (Bhatara et al. 2016).

**TABLE 1:** The eight emotions in an arousal‐valence grid (after Goudbeek and Broersma 2010, 2212), with basic emotions marked with ‘*’.

		Valence	
		Positive	Negative
Arousal	High	Joy*	Anger*
		Pride	Fear*
	Low	Tenderness	Sadness*
		Relief	Irritation

### 2.3 Procedure

**FIGURE 1:**
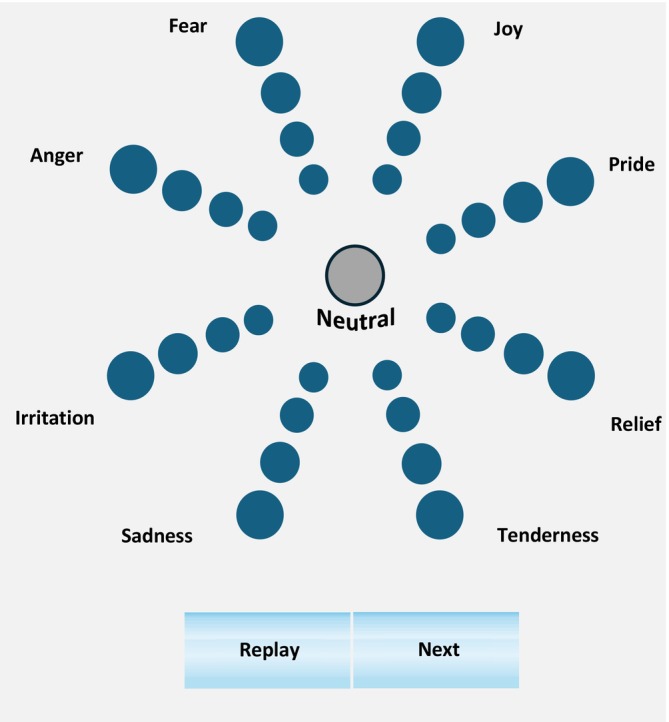
The emotion wheel in English (adapted from Liang et al. 2023). Translation in Dutch and Korean, Joy: “Blijdschap”, “행복”; Pride: “Trots”, “자랑스러움”; Relif: “Opluchting”, “안도감”; Tenderness: “Vertedering”, “애정”; Sadness: “Verdriet”, “슬픔”; Irritation: “Irritatie”, “짜증”; Anger: “Woede”, “분노”; Fear: “Angst”, “공포”; Neutral: “Neutral”, “중립”.

Participants completed the experiment individually in a sound‐attenuated booth at Radboud University in the Netherlands or at the University of Seoul in South Korea. The emotion wheel, with eight emotions and four circles in different sizes indicating different levels of intensity (Figure 1), was displayed on the computer screen with buttons labeled in the participants' native language (Dutch or Korean). Participants listened to the recordings via high‐quality headphones. The whole experiment was administered via JavaScript on a standard laboratory computer and took approximately 35–45 min.

All participants were given instructions in their native language. Participants were asked to listen to each stimulus and to select the target emotion from the eight options shown on the screen. The next step was to rate the emotional intensity they thought the stimulus conveyed. As a last option, they could choose Neutral (no category, no emotional intensity). Participants could listen to each stimulus more than once if they wished. In the previous study, we investigated only the first categorical response given. In the present study, we focused on the intensity ratings.

The whole experiment included two parts, blocked by language, with 128 stimuli per language. The experiment started with the first part of the Korean stimuli. Before the experiment, there was a practice session, and participants were informed about the language they would hear.

### 2.4 Statistical Analyses

The data analyses were performed in R (R Core Team, 2022). To address all hypotheses, we conducted a series of linear mixed‐effects models with the *lme4* package (Bates et al. 2015), and pairwise comparisons for Hypotheses 1 and 2.

For Hypotheses 1 and 2, we performed separate analyses for the Dutch and Korean listener groups, as their in‐group bias, if present, works the other way around with respect to the Dutch and Korean speakers and should be treated as an independent group effect. Each group of listeners should show its group‐specific bias. The analyses included two fixed predictors: Speaker Language (Dutch vs. Korean recordings) and Emotion (Joy, Pride, Anger, Fear, Tenderness, Relief, Sadness, and Irritation). The outcome variable was Intensity, which is a continuous variable. Additionally, we included two random intercepts (Listener and Speaker) in all analyses. Random slopes with Emotion involved systematically returned non‐convergent models. Therefore, we added only random by‐listener slopes for Speaker Language. As for the fixed effects, the interaction between Speaker Language and Emotion was significant in all analyses, and removing it always led to higher AICs. Therefore, we kept the interaction effect. To investigate the in‐group bias per Emotion, we completed the analyses by pairwise comparisons using the package (EMMEANS). The statistical summaries are given in Appendix A. Models 1 and 2 cover all responses, and Models 3 and 4 cover the correct responses.

For Hypotheses 3–5, the analyses included three fixed predictors: Speaker Language, Listener Language, plus one of the binary distinctions: Arousal (high‐arousal vs. low‐arousal emotions)/Valence (negative vs. positive emotions)/Basicness (basic vs. non‐basic emotions). Again, we repeated the analysis: first for all responses, then for correct responses only. Consequently, we have 3 (the three binary distinctions) × 2 (all responses, correct responses) analyses. Their statistical summaries can be found in Models 5–10 in Appendix A. The random parts of these models were treated in the same way. We included all three random intercepts and the interaction slopes with three fixed variables. In Model 6, one slope was removed because there was no random variation. We preferred to keep all interactions between the three fixed variables in the analysis to ensure the outcomes are directly comparable. Reducing the models did not return deviant estimates of the effects involved. Our final step in these analyses was to add Emotion as a random effect to visualize how individual emotions deviated from the group they were assigned. Again, this step did not change the estimates of the fixed effects of the overall model.

All linear mixed‐effects models used regression‐style contrast coding for predictors with two levels (−0.5 and 0.5 contrast coding for the first and second levels shown above).

## 3 Results

### 3.1 The In‐Group Bias in Intensity Ratings Across All Responses (Hypothesis 1)

**FIGURE 2:**
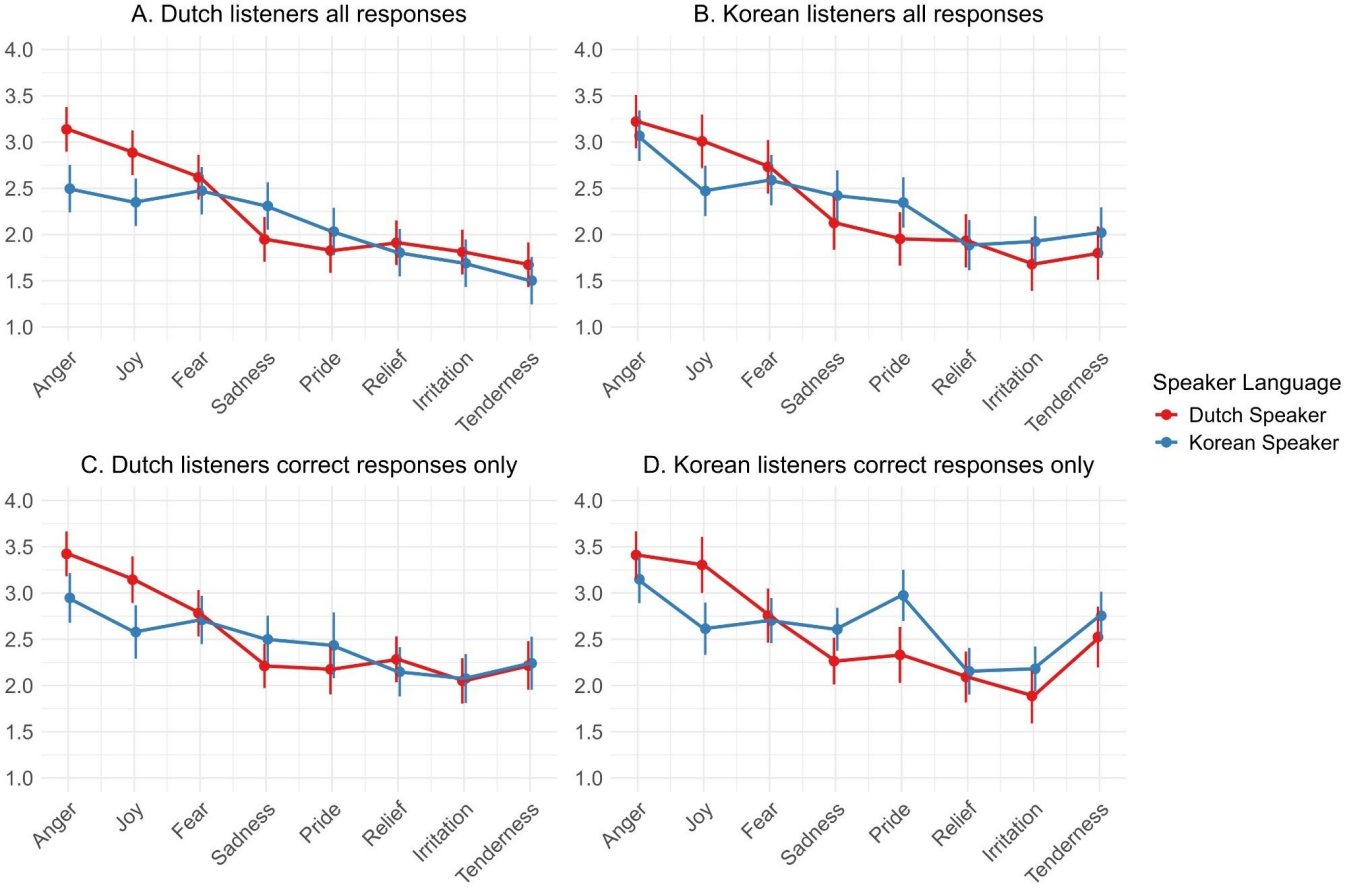
Intensity ratings by Dutch and Korean listeners (a) across all responses (correct and incorrect responses), (b) across the correct responses. Error bars represent ±2 SE in all figures.

The first research question examined whether there exists an in‐group bias in intensity ratings across all responses. We hypothesized that both listener groups would rate emotions produced in their native language as higher in intensity than those expressed in the unknown language across all responses (Hypothesis 1). This hypothesis was tested in Models 1 and 2, which examined intensity ratings of the eight emotions aggregated across correct and incorrect responses, separately for Dutch and Korean listeners. Therefore, we split the entire dataset into two subsets based on the listeners' language. Each model included two fixed predictors: Speaker Language and Emotion, and random intercepts for listeners and speakers, as well as random by‐listener slopes for Speaker Language, which improved model fit significantly, resulting in the lowest AIC score. The random‐effects structure showed that intensity ratings varied across listener and speaker groups, highlighting individual differences in ratings. The mean scores and their confidence intervals are given in Figure 2A,B. In both panels, we see that the two connecting lines tend to go down, but neither line (red or blue) is consistently higher than the other. They cross each other.

#### 3.1.1 Intensity Ratings of Emotions in the Whole Dataset by Dutch Listeners

The results of Model 1 (see Appendix A) revealed a significant main effect of Speaker Language on Intensity (Δ = 0.64), favoring Dutch speakers. Furthermore, there was a significant main effect of Emotion on intensity, such that intensity ratings were higher for Anger than for any other emotion by Dutch listeners across all responses. More importantly, there were six significant two‐way interactions between Speaker Language and Emotion (Pride/Fear/Tenderness/Relief/Sadness/Irritation), indicating that intensity ratings differed across speaker languages and emotions. To further examine the specific differences in intensity ratings for each emotion between Dutch and Korean recordings, we performed detailed Estimated Marginal Means (EMMEANS) analyses for pairwise comparisons across the eight emotions between Dutch and Korean recordings. To avoid Type I error caused by multiple comparisons, we used the *Tukey* adjustment. The results demonstrated that Dutch listeners gave significantly higher intensity ratings to Anger and Joy in Dutch than in Korean recordings, whereas they gave higher intensity ratings to Sadness in Korean than in Dutch recordings (see Table 2).

**TABLE 2:** Summary of EMMEANS analyses for Dutch listeners across all responses (standard error in parentheses, in all tables; significant *p*‐values in boldface).

Emotion	Speakers		df	*t*	*p*
	Dutch	Korean			
Anger	3.14 (0.12)	2.50 (0.13)	20.10	3.91	**0.001**
Joy	2.89 (0.12)	2.35 (0.13)	20.10	3.27	**0.004**
Pride	1.83 (0.12)	2.03 (0.13)	20.10	−1.25	0.224
Fear	2.62 (0.12)	2.47 (0.13)	20.10	0.89	0.387
Tenderness	1.67 (0.12)	1.50 (0.13)	20.10	1.05	0.309
Relief	1.91 (0.12)	1.80 (0.13)	20.10	0.65	0.522
Sadness	1.95 (0.12)	2.31 (0.13)	20.10	−2.20	**0.040**
Irritation	1.81 (0.12)	1.69 (0.13)	20.10	0.74	0.469

#### 3.1.2 Intensity Ratings of Emotions in the Whole Dataset by Korean Listeners

The results of Model 2 (see Appendix A) demonstrated no significant main effect of Speaker Language on intensity, as Korean listeners gave slightly different intensity ratings on average for emotions regardless of the type of recordings (Δ = 0.15), but this difference was not significant. Furthermore, there was a significant main effect of Emotion on intensity, indicating that intensity ratings varied across emotions. Additionally, there were five significant two‐way interactions between Speaker Language and Emotion (Joy/Pride/Tenderness/Sadness/Irritation), indicating that Korean listeners gave different intensity ratings to emotions across Dutch and Korean recordings. EMMEANS analyses (Table 3) showed that Korean listeners gave significantly higher intensity ratings to Pride in Korean than in Dutch recordings, while they gave higher intensity ratings to Joy in Dutch than in Korean recordings.

**TABLE 3:** Summary of EMMEANS results for Korean listeners across all responses.

Emotion	Speakers		df	*t*	*p*
	Dutch	Korean			
Anger	3.22 (0.15)	3.07 (0.14)	21.90	0.90	0.380
Joy	3.01 (0.15)	2.47 (0.14)	21.90	3.13	**0.005**
Pride	1.95 (0.15)	2.35 (0.14)	21.90	−2.29	**0.032**
Fear	2.73 (0.15)	2.59 (0.14)	21.90	0.84	0.413
Tenderness	1.80 (0.15)	2.02 (0.14)	21.90	−1.29	0.210
Relief	1.93 (0.15)	1.89 (0.14)	21.90	0.27	0.787
Sadness	2.12 (0.15)	2.42 (0.14)	21.90	−1.73	0.097
Irritation	1.68 (0.15)	1.92 (0.14)	21.90	−1.43	0.168

Together, Dutch and Korean listeners displayed similar patterns in intensity ratings for the eight emotions across Dutch and Korean recordings, such that they gave higher intensity ratings to Anger, Joy, Fear, and Relief in Dutch than in Korean recordings, whereas they gave higher intensity ratings to Pride and Sadness in Korean than in Dutch recordings. However, both listener groups gave slightly higher intensity ratings to Tenderness and Irritation in their native language than in the unknown language, although this interaction did not reach statistical significance.

All in all, Hypothesis 1 has to be rejected. Neither listener group gave consistently higher intensity ratings to emotions produced in their native language than in the unknown language. Instead, they gave higher intensity ratings to particular emotions, even when these emotions were produced in the unknown language.

### 3.2 The In‐Group Bias in Intensity Ratings Across Correct Responses (Hypothesis 2)

As shown in Figure 2, both listener groups gave higher intensity ratings to correct responses than to all responses, indicating that intensity ratings are higher when emotions were recognized. More importantly, Panels C and D in Figure 2 show again two crossing lines for the correct responses. The second hypothesis focuses on the presence of an in‐group bias in the correct responses. Again, we performed two analyses for the two listener groups. We applied the same analyses as for all responses.

#### 3.2.1 Intensity Ratings of Emotions in the Subset of Correct Responses by Dutch Listeners

The results of Model 3 (see Appendix A) demonstrated a significant main effect of Speaker Language on intensity, as Dutch listeners gave higher intensity ratings to emotions produced in Dutch than in Korean recordings (Δ = 0.48). Also, there was a significant main effect of Emotion (Joy/Pride/Fear/Tenderness/Relief/Sadness/Irritation) on intensity, such that intensity ratings were higher for Anger than for any other emotions. Importantly, there were significant two‐way interactions between Speaker Language and Emotion (Pride/Fear/Tenderness/Relief/Sadness/Irritation), suggesting that intensity ratings varied across emotions and speaker languages. Subsequent EMMEANS analyses (Table 4) revealed that Dutch listeners gave higher intensity ratings to Anger, Joy, Fear, and Relief in Dutch than in Korean recordings, while they gave higher intensity ratings to Pride, Tenderness, Sadness, and Irritation in Korean than in Dutch recordings. However, the difference between Korean and Dutch recordings reached statistical significance only for Anger and Joy.

**TABLE 4:** Summary of EMMEANS analyses for Dutch listeners in the correct responses.

Emotion	Speakers		df	*t*	*p*
	Dutch	Korean			
Anger	3.42 (0.12)	2.95 (0.14)	22.90	2.84	**0.009**
Joy	3.14 (0.13)	2.58 (0.15)	30.50	3.14	**0.004**
Pride	2.18 (0.14)	2.43 (0.18)	61.70	−1.21	0.232
Fear	2.78 (0.13)	2.71 (0.13)	23.10	0.43	0.670
Tenderness	2.22 (0.13)	2.24 (0.15)	32.40	−0.14	0.894
Relief	2.28 (0.13)	2.15 (0.14)	23.70	0.80	0.431
Sadness	2.21 (0.12)	2.50 (0.13)	19.40	−1.80	0.088
Irritation	2.05 (0.13)	2.08 (0.14)	22.70	−0.15	0.883

#### 3.2.2 Intensity Ratings of Emotions in the Subset of Correct Responses by Korean Listeners

The results of Model 4 (see Appendix A) revealed no significant main effect of Speaker Language on Intensity (Δ = 0.26). Also, there was a significant main effect of Emotion on intensity, such that intensity ratings were higher for Anger than for any other emotion. Additionally, there were six significant two‐way interactions between Speaker Language and Emotion (Joy/Pride/Tenderness/Relief/Sadness/Irritation). We performed Estimated Marginal Means (EMMEANS) analyses to further examine the differences in intensity ratings for each emotion across Dutch and Korean recordings (Table 5). The results demonstrated that Korean listeners gave higher intensity ratings to Anger, Joy, and Fear in Dutch than in Korean recordings, whereas they gave higher intensity ratings to Pride, Tenderness, Relief, Sadness, and Irritation in Korean than in Dutch recordings. However, only the ratings for Joy, Pride, and Sadness reached statistical significance.

**TABLE 5:** Summary of EMMEANS analyses for Korean listeners in the correct responses.

Emotion	Speakers		df	*t*	*p*
	Dutch	Korean			
Anger	3.41 (0.13)	3.15 (0.13)	37.30	1.64	0.109
Joy	3.30 (0.15)	2.61 (0.15)	72.00	3.66	**0.001**
Pride	2.33 (0.15)	2.97 (0.14)	68.50	−3.45	**0.001**
Fear	2.76 (0.15)	2.70 (0.13)	46.60	0.31	0.757
Tenderness	2.52 (0.17)	2.75 (0.13)	76.30	−1.20	0.234
Relief	2.09 (0.14)	2.15 (0.13)	42.20	−0.37	0.710
Sadness	2.26 (0.13)	2.61 (0.12)	26.90	−2.34	**0.027**
Irritation	1.89 (0.15)	2.18 (0.12)	47.40	−1.73	0.090

All in all, Hypothesis 2 is rejected. Neither listener group gave consistently higher intensity ratings to emotions produced in their native language than in the unknown language, even when they recognized the emotion. However, both listener groups gave higher intensity ratings to certain emotions, although these emotions were produced in the unknown language.

### 3.3 The Effect of Arousal on Intensity Ratings (Hypothesis 3)

The third hypothesis concerns the effect of Arousal on intensity ratings, both across all responses and correct responses. The hypothesis was that both listener groups would give higher intensity ratings to high‐arousal than to low‐arousal emotions across all responses and especially across correct responses (Hypothesis 3). The statistical results of the mixed‐effects analyses are given in Appendix A, Model 5 (all responses) and Model 6 (correct responses). The predicted outcomes are visualized in Figure S1.

Panels A and B exhibit a quite similar pattern, but Panel B shows higher overall scores. More importantly, the outcomes show a clear split between high‐arousal and low‐arousal emotions. The main effect of arousal is 0.67 in the left panel (all responses) and 0.68 in the right panel (correct responses). Two interactions are significant as well. In Panel A, it is the interaction between Speaker Language and Listener Language. In Panel B, it is the interaction between Speaker Language and Arousal.

Panel A shows that Dutch listeners gave 0.29 higher ratings to emotions produced in Dutch than to those produced in Korean, whereas Korean listeners gave 0.07 higher ratings to emotions produced in Korean than to those produced in Dutch. However, there was no three‐way interaction between Speaker Language, Listener Language, and Arousal, indicating that the two‐way interaction between Speaker Language and Listener Language was not modulated by Arousal.

In the subset of correct responses only, there was a significant two‐way interaction between Speaker Language and Arousal, such that the intensity rating was 0.67 higher for high‐arousal emotions produced in Dutch than in Korean, whereas both listener groups gave 0.36 higher ratings to low‐arousal emotions produced in Korean than in Dutch.

**FIGURE 3:**
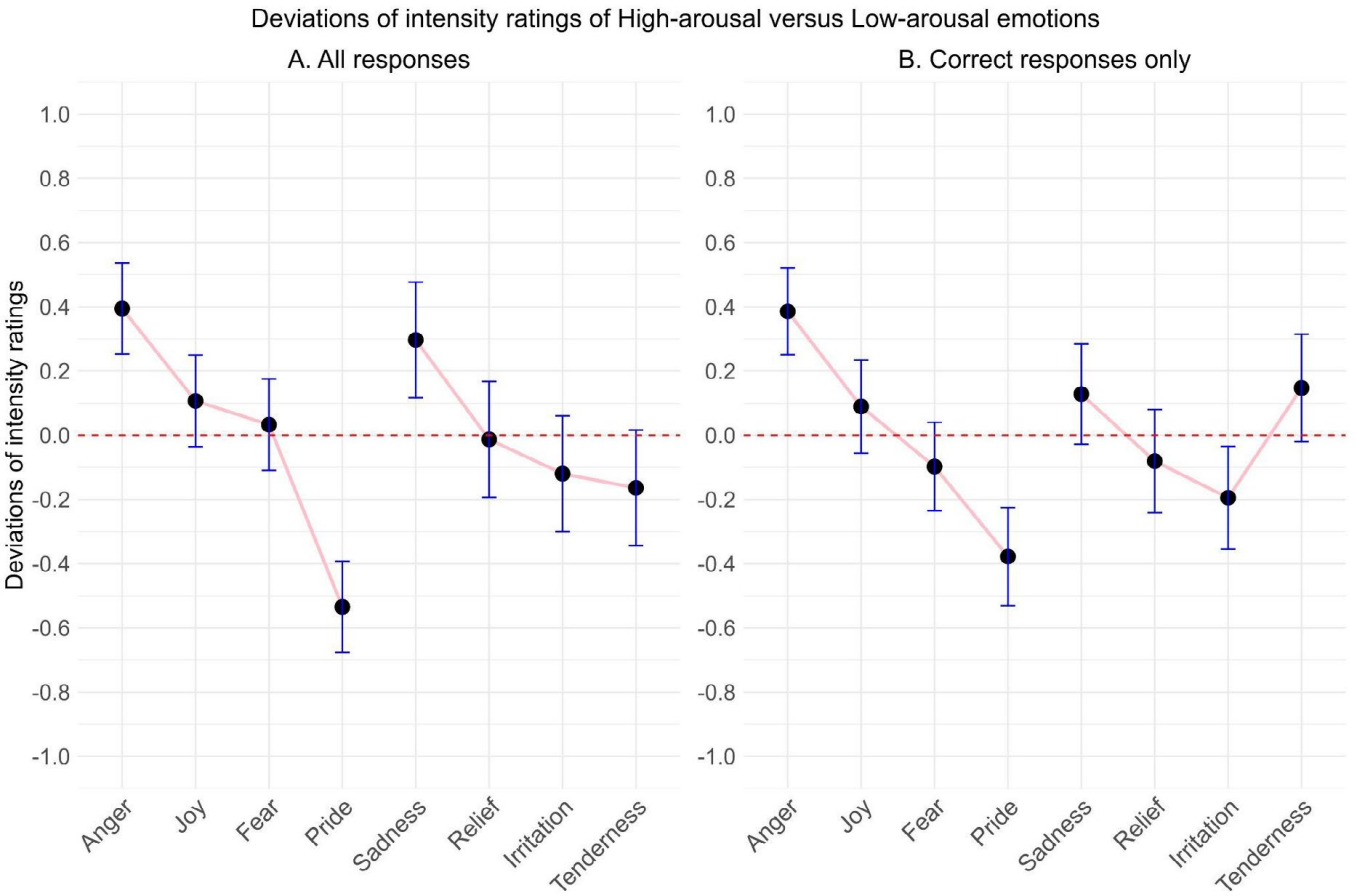
Intercepts of the intensity ratings of high‐arousal (Anger, Joy, Fear, Pride) and low‐arousal emotions (Sadness, Relief, Irritation, Tenderness) (A) across all responses, (B) across the correct responses, with their confidence interval (2SE). The order of emotions from left to right is listed as follows: (1) the four high‐arousal emotions are presented left, and the four low‐arousal emotions are presented right; (2) within each group (high‐arousal or low‐arousal), emotions are listed according to the size of their intercepts in the analysis of all responses (from high to low). For numerical values of intercepts in Panels A and B, see Appendix B.

Overall, the effect of arousal is obviously significant and by far the strongest one in the analysis. On the other hand, the *R* ^2^ values given in Figure S1 predict a substantial part (0.234–0.235) of the variance present in the fixed effects (the conditional *R* ^2^). This effect is far from perfect, but including the random effects substantially reduces the effect size (unconditional *R* ^2^): 0.100 in Panel A, 0.140 in Panel B. Since the effect is medium‐sized, we should examine variation in emotions. The success of the split can be investigated by including emotions within their categories, enabling evaluation of their contribution. These intercepts are visualized in Figure 3, along with their confidence intervals (high‐arousal emotions on the left and low‐arousal emotions on the right). Within each group of four emotions, the mean is zero by definition. The difference between high‐ and low‐arousal emotions was already captured by the main effect of arousal (Δ = 0.67 and 0.68). If the emotions are random effects within their categories, their confidence intervals would include zero. Anger is extremely high, and Pride is extremely low within both panels of Figure 3. Sadness is too high in the left panel in relation to the other three low‐arousal emotions. Pride is about −0.52, a value that is almost the difference between high‐ and low‐arousal emotions. It is a clear violation of the predicted effect of high‐arousal emotions. The same can be said of Sadness in Panel A regarding low‐arousal emotions. These results indicate that although intensity ratings are positively related to the level of arousal as predicted by Hypothesis 3, the binary split between high‐arousal versus low‐arousal does not yield an across‐the‐board dichotomy of the eight emotions in terms of intensity.

### 3.4 The Effect of Valence on Intensity Ratings (Hypothesis 4)

The fourth hypothesis concerns the effect of Valence on intensity ratings, both across all responses and correct responses. The hypothesis was that both listener groups would rate negative emotions as more intense than positive emotions across all responses, especially in correct responses (Hypothesis 4). The statistical results of the mixed‐effects analysis are given in Appendix A, Model 7 (all responses) and Model 8 (correct responses). The predicted outcomes are visualized in Figure S2.

Panels A and B show a similar pattern, with higher intensity ratings in Panel B. Notably, the outputs exhibit a clear split between negative and positive emotions. The main effect of valence is 0.30 in the left panel (all responses) and 0.17 in the right panel (correct responses). In Panel A, there is a significant two‐way interaction between Speaker Language and Listener Language. In Panel B, there is a significant three‐way interaction between Speaker Language, Listener Language, and Valence.

Panel A demonstrates that Dutch listeners gave 0.29 higher ratings to emotions produced in Dutch than to those produced in Korean, whereas Korean listeners gave only slightly higher ratings (Δ = 0.07) to emotions produced in Korean than to those produced in Dutch. However, the three‐way interaction between Speaker Language, Listener Language, and Valence did not reach statistical significance, revealing that the two‐way interaction between Speaker Language and Listener Language was not modulated by Valence.

In the subset of correct responses, there was no significant two‐way interaction between Speaker Language and Listener Language. However, there was a significant three‐way interaction between Speaker Language, Listener Language, and Valence. Specifically, both listener groups gave slightly higher intensity ratings to negative emotions produced in Dutch than in Korean (Δ for Dutch listeners: 0.03; Δ for Korean listeners: 0.08). However, both listener groups gave higher intensity ratings to positive emotions produced in their native language than in the unknown language (Δ for Dutch listeners: 0.32; Δ for Korean listeners: 0.08).

**FIGURE 4:**
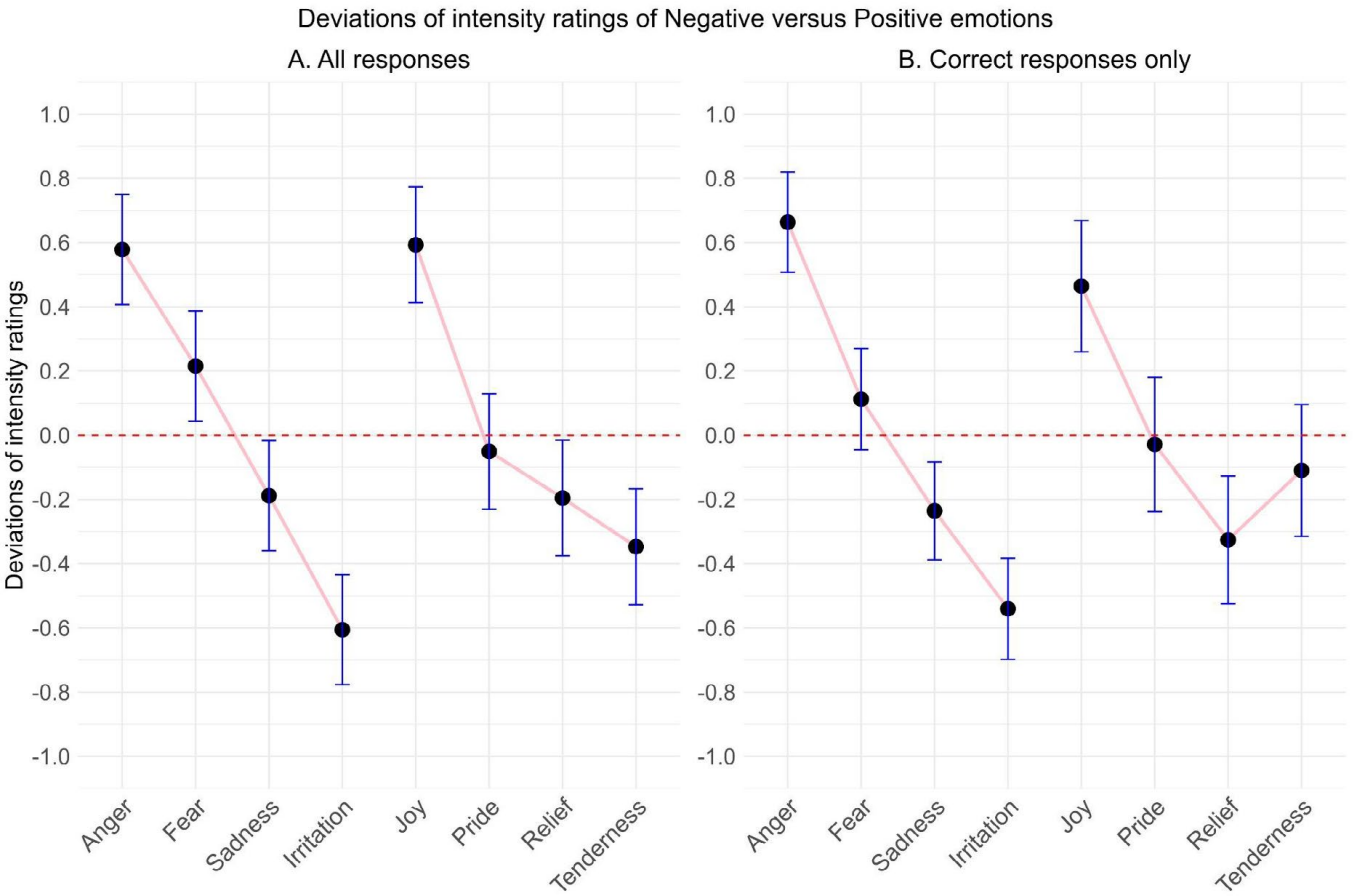
Intercepts of the intensity ratings of negative (Anger, Fear, Sadness, Irritation) and positive emotions (Joy, Pride, Relief, Tenderness) (A) across all responses, (B) across the correct responses, with their confidence interval (2SE). The order of emotions from left to right is listed as follows: (1) the four negative emotions are presented left, and the four positive emotions are presented right; (2) within each group (negative or positive), emotions are listed according to the size of their intercepts in the analysis of all responses (from high to low). For numerical values of intercepts in Panels A and B, see Appendix B.

Together, the effect of valence is significant but weaker than that of the split by Arousal. Moreover, the *R* ^2^ values given in Figure S2 account for only a small portion of the variance shown in the fixed effects (the conditional *R* ^2^), but including the random effects results in much lower values: 0.028 in Panel A and 0.011 in Panel B. Since the effect was small, we further examined differences in emotions within the negative and positive emotion categories. It is possible to assess their relative contribution by examining the intercepts of each emotion within its category. The results for these intercepts are visualized in Figure 4, along with their confidence intervals (negative emotions on the left, and positive emotions on the right).

By definition, the mean of each group of four emotions is set to zero. The difference between negative and positive emotions was shown by the main effect of valence (Δ = 0.30 and 0.17). When emotions are treated as random effects within their categories, their confidence intervals should include zero. Anger is the second highest in Panel A and the highest in Panel B of Figure 4, while Irritation is the lowest in both panels of Figure 4. Joy is too high in the left panel compared to the other three positive emotions. Irritation is around −0.60, a value that is almost twice the difference between negative and positive emotions. It is an obvious violation of the predicted effect of negative emotions. The same can be said of Joy in Panel A regarding positive emotions. These results reveal that, although intensity ratings are positively correlated with valence as predicted by Hypothesis 4, the binary split between negative and positive does not yield a clear dichotomy in intensity ratings across the eight emotions.

### 3.5 The Effect of Basicness on Intensity Ratings (Hypothesis 5)

The fifth hypothesis concerns the effect of Basicness on intensity ratings, both across all responses and correct responses. Both listener groups were expected to give higher intensity ratings to basic than to non‐basic emotions across all responses, especially to correct responses (Hypothesis 5). This hypothesis was addressed with models 10 and 11. The statistical results of the mixed‐effects analysis are given in Appendix A, Model 9 (all responses) and Model 10 (correct responses). The predicted outcomes are visualized in Figure S3.

Panels A and B exhibit similar patterns, with slightly higher intensity ratings in Panel B. Importantly, they reveal a clear split between basic and non‐basic emotions. The main effect of Basicness is 0.75 in the left panel and 0.54 in the right panel. In Panel A, there is a significant two‐way interaction between Speaker Language and Listener Language. In Panel B, there is a significant two‐way interaction between Speaker Language and Listener Language.

Dutch listeners (Panel A) gave 0.29 higher intensity ratings to emotions produced in Dutch than in Korean, whereas Korean listeners gave slightly higher ratings to emotions produced in Korean than in Dutch (Δ = 0.07). Furthermore, there was a significant three‐way interaction between Speaker Language, Listener Language, and Basicness, indicating that Basicness modulated the two‐way interaction between Speaker Language and Listener Language.

Panel B shows a significant two‐way interaction between Speaker Language and Listener Language. Here, both listener groups gave higher ratings to emotions produced in their native language than in the unknown language, with 0.19 and 0.15 higher in‐group than out‐group intensity ratings for Dutch and Korean listeners, respectively. More importantly, there was a significant three‐way interaction between Speaker Language, Listener Language, and Basicness. Specifically, Dutch listeners gave 0.19 higher ratings to basic emotions in Dutch than in Korean, while they gave slightly higher ratings (Δ = 0.01) to non‐basic emotions in Dutch than in Korean. Korean listeners gave 0.12 higher ratings to basic emotions in Dutch than in Korean, whereas they gave 0.27 higher ratings to non‐basic emotions in Korean than in Dutch.

**FIGURE 5:**
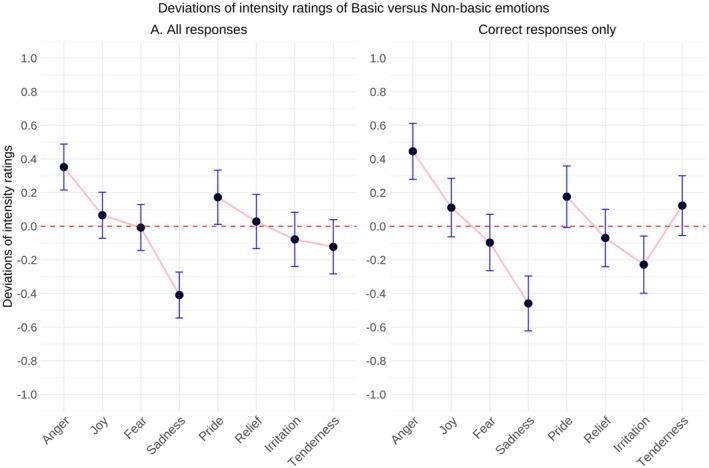
Intercepts of the intensity ratings of basic (Anger, Joy, Fear, Sadness) and non‐basic emotions (Pride, Relief, Irritation, Tenderness) (A) across all responses, (B) across correct responses, with their confidence interval (2SE). The order of emotions from left to right is listed as follows: (1) the four basic emotions are presented left, and the four non‐basic emotions are presented right; (2) within each group (basic or non‐basic), emotions are listed according to the size of their intercepts in the analysis of all responses (from high to low). For numerical values of intercepts in Panels A and B, see Appendix B.

Altogether, the effect of basicness is statistically significant and strong in the analysis. Moreover, the *R* ^2^ values presented in Figure S3 account for a considerable part of the variance: 0.258 and 0.163 of the variance shown in the fixed effects (the conditional *R* ^2^), but including the random effects leads to lower results: 0.121 in Panel A and 0.087 in Panel B. Given that the effect is medium‐sized, we further investigated variations in emotions across the basic and non‐basic emotion categories. The success of the split can be analyzed by incorporating emotion as a random effect, allowing an evaluation of their relative contributions by examining the intercepts of individual emotions within each category. These intercepts are visualized in Figure 5, along with their confidence intervals (basic emotions on the left and non‐basic emotions on the right). The mean within each group of four emotions is defined as zero by definition.

The difference between basic and non‐basic emotions was reflected in the main effect of basicness (Δ = 0.75 and 0.54). When emotions are treated as random effects within their categories, their confidence intervals would include the zero value. Anger is the highest, and Sadness is the lowest within both panels of Figure 5. Pride is too high in the left panel compared to other non‐basic emotions. Sadness is at about −0.40, a value that is close to the difference between basic and non‐basic emotions. Clearly, it violates the predicted effect of basic emotions. These results suggest that although intensity ratings are positively related to basicness overall, the binary split between basic and non‐basic emotions does not yield a clear dichotomy in intensity for the eight emotions.

### 3.6 Analyses of Arousal, Valence, and Basicness Compared

In Table 6, we compare the statistical outcomes of the three binary splits presented above to evaluate their relative success in predicting emotional intensity ratings.

**TABLE 6:** Summary of the analysis of the dimensions of Arousal, Valence, and Basicness across all responses and correct responses only; violations are intercepts of emotions with an outlying value in the wrong direction.

Dimension	Data	*R* ^2^		Effect size [CI]	Violations
		Marginal	Conditional		
Arousal	All	0.098	0.235	−0.67 [−0.83, −0.51]	2
	Correct only	0.141	0.234	−0.68 [−0.84, −0.52]	1
Valence	All	0.028	0.084	−0.30 [−0.44, −0.16]	3
	Correct only	0.011	0.087	−0.17 [−0.33, −0.01]	3
Basicness	All	0.121	0.258	−0.75 [−0.93, −0.57]	2
	Correct only	0.087	0.163	−0.54 [−0.74, −0.34]	1

The results revealed that conditional *R* ^2^ was higher, of course, than marginal *R* ^2^ in each dimension, indicating that the combination of both fixed predictors and random effects explains the variance of intensity ratings better than fixed predictors alone, but the values of 0.084 and 0.087 are quite low for Valence. None of the three dimensions perfectly dichotomizes intensity. The higher conditional *R* ^2^ values are medium‐sized. Furthermore, we found that Arousal and Basicness had clearly higher conditional *R* ^2^ values than Valence, with the highest value for Basicness across all responses (0.258) and the highest for Arousal across correct responses (0.234), indicating that intensity ratings were more strongly related to Arousal and Basicness than to Valence. Also, Valence shows more deviant emotions than Arousal and Basicness, suggesting that Valence's impact on intensity ratings is minimal. This finding corroborates earlier findings that arousal is positively related to intensity (Laukka et al. 2005). All three splits showed the predicted effect; however, none of the dimensions yielded an across‐the‐board split between the emotions.

## 4 Discussion

This study investigated intensity ratings of vocal emotions with a balanced design involving speakers and listeners from typologically different cultures and languages; that is, Dutch and Korean. Our results partially corroborate earlier findings and shed new light on the concept of intensity in the affective science. Prior studies on intensity ratings have predominantly focused on facial expressions (Ekman et al. 1987; Kommattam et al. 2019; Matsumoto and Ekman 1989; Shioiri et al. 1999; Yrizarry et al. 1998). Some studies investigated intensity ratings in the vocal domain, but not cross‐culturally (Holz et al. 2021; Juslin and Laukka 2001; Laukka et al. 2005). Most of these studies target basic emotions only (Holz et al. 2021; Juslin and Laukka 2001; Laukka et al. 2005), and/or studied an unbalanced number of emotions in terms of arousal and valence. Consequently, the current literature on emotional intensity cannot be generalized to non‐basic emotions. In our study, therefore, we included four basic emotions (Joy, Anger, Fear, and Sadness) and four non‐basic emotions (Pride, Tenderness, Relief, and Irritation), which are balanced in arousal and valence, making it possible to examine the relative contributions of arousal, valence, and basicness to intensity ratings. This would be the first study to explore intensity ratings of vocal emotions along three dimensions: arousal, valence, and basicness.

This study had two main goals. First, we tested the in‐group bias in intensity ratings across all responses (Hypothesis 1) and then in the subset of correct responses only (Hypothesis 2). The notion of in‐group bias entails that individuals rate facial expressions of emotion produced by members of their own (or similar) ethnic group as more intense than expressions produced by members of a different group (Kommattam et al. 2019). The in‐group bias was attested for facially expressed emotions, but it is unclear whether the principle also applies to vocal expressions of emotion. First, our results contradicted the in‐group bias hypothesis, as neither listener group gave higher intensity ratings to emotions produced in their native language than to those produced in the unknown language (Hypothesis 1). Rather, intensity ratings were higher for some specific emotions, even when these emotions were produced in the unknown language. Instead of in‐group bias, intensity ratings are more emotion‐dependent, which affects the degree of perceived intensity. For instance, Dutch listeners exhibited an in‐group bias for emotions like Anger and Joy, whereas Korean listeners showed a bias for these two emotions but not an in‐group bias, as they rated Anger and Joy as more intense in Dutch than in Korean. Possibly, then, some emotions may be produced with more intensity than others in all (or at least a majority of) languages and/or cultures.

Second, we tested in‐group bias in intensity ratings only for correct responses. Similar to Hypothesis 1, we found no general in‐group bias in intensity ratings in this subset (Hypothesis 2). Here too, both listener groups gave higher intensity ratings to Anger and Joy in Dutch than in Korean, whereas they gave higher ratings to Sadness in Korean than in Dutch. These findings provide evidence against a general in‐group bias in intensity ratings, even when observations are restricted to correct responses only. Again, listeners rated some particular emotions higher, even though they were not produced in their native language.

The in‐group intensity bias was originally formulated and tested in the context of cross‐cultural perception of facial expressions of emotion. We adopted the in‐group intensity bias hypothesis from Kommattam et al. (2019). These authors present a survey of cross‐cultural perception of emotional intensity for three groups of white European speakers and listeners: Dutch, English, and Finnish. The first nine studies investigated the perception of a single emotion; that is, embarrassment—a non‐basic emotion. Three more studies were reported in the survey; these tested nine different emotions but reported aggregated results across all nine without providing a breakdown by specific emotion. It is impossible, therefore, to verify whether indeed all nine emotions consistently showed the in‐group bias or whether one or more emotions deviated from the general effect. Moreover, the 12 studies reported by Kommattam and associates presented facial expressions of emotion produced by white (Dutch, American) versus Arab (Moroccan, Turkish) models, with perceivers exclusively white Europeans (or European Americans). The complementary condition, with observers from an Arab background, was not implemented, so that it is possible that Arab observers, had they been included in the experiments, might also have judged the European expressions of emotion more intense than the same emotions expressed by fellow Arabs. To address this problem, we adopted a “two‐by‐two” design by involving both speakers and listeners whose cultures and languages are typologically different. This fully cross‐cultural design enables us to effectively test in‐group bias in intensity ratings. Finally, we cannot exclude the possibility that the facial and vocal transmission channels differ fundamentally, so that effects reliably observed in the facial modality may be absent or even reversed in the vocal modality. Interestingly, in‐group bias was strongest for facially expressed non‐basic emotions (contempt, pride, embarrassment). This finding supports the idea that secondary emotions are expressed less clearly than basic emotions, so that only an in‐group perceiver can pick up the cues. Interpreting emotions was found to be more challenging from vocal than from visual cues (App et al. 2011). Its absence (or emotion specificity) in the vocal domain may be attributed to the complexity and strength of the vocal modality (e.g., the audible differences of sound features even across languages). Despite this, the in‐group intensity bias was less clear for weakly expressed vocal emotions than for strongly expressed ones in the recent study by Zhang and Pell (2022), and the in‐group bias was not consistently found across all four emotions tested (see the summary on p. 6). Our results confirm Zhang and Pell (2022) insofar as the in‐group intensity bias did not apply to all emotions tested. This raises the question of whether the in‐group intensity bias operates through the same mechanism in facial and vocal emotion perception.

Further, we observed that both listener groups rated certain vocal emotions more intensely when produced in the unknown language than in their native language. This pattern was inconsistent, depending on the emotion and the speaker's language. It can probably be attributed to the fact that Dutch actors expressed Anger more intensely in Dutch than Korean actors did in Korean. This highlights the need to ensure “stimulus equivalence” (Matsumoto 2002) when testing the in‐group bias cross‐culturally. Differences caused by language‐specific prosodic features may override any potential in‐group bias.

The second goal of our study was to examine the role of arousal, valence, and basicness, as binary properties of emotional expressions, in intensity ratings across all responses and for correct responses only (Hypotheses 3–5). Given the relatively larger number of eight emotions distributed across these three dimensions, our results provide new insight into their relative contributions to intensity ratings.

First, high‐arousal emotions were rated as more intense than low‐arousal emotions (Hypothesis 3), which confirms earlier studies (Holz et al. 2021; Laukka et al. 2005). The level of arousal is closely related to the rated intensity of emotions, which share similar acoustic cues, such as fundamental frequency (F0), fundamental frequency variation, and speech rate. (Laukka et al. 2005).

Second, negative emotions were rated as more intense than positive emotions (Hypothesis 4). Little is known about the relationship between valence and intensity, although it was found earlier that non‐verbal vocalizations with high and peak intensities were more prone to be rated as negative emotions (Holz et al. 2021). Notably, Holz et al. (2021) asked participants to rate the dimensions of emotions by presenting the dimensions (i.e., arousal and valence). In their experiment, these dimensions were neither mentioned nor rated in our study. Our study examined the role of valence, as a binary characteristic (assigned to emotions on theoretical grounds), in intensity ratings. The difference in intensity ratings we found between negative and positive emotions may be caused by the fact that negative emotions are more related to threats and dangers than positive emotions (Shiota et al. 2004). The fact that negative emotions tend to be produced with higher intensity than positive emotions would be beneficial for survival.

Third, basic emotions were rated as more intense than non‐basic emotions (Hypothesis 5). Ours is arguably the first study comparing intensity ratings between basic and non‐basic emotions in vocal emotions, although Kommattam et al. (2019) included three non‐basic emotions (secondary emotions) in intensity ratings of facial emotions. In this study, we included four basic (anger, fear, joy, sadness) and four non‐basic emotions (irritation, pride, relief, tenderness). However, because the classic set of six basic emotions is unbalanced in terms of positive and negative emotions, we could not balance the four basic emotions (anger, fear, happiness, and sadness) in terms of arousal and valence, resulting in a non‐orthogonal design in basicness. The high‐intensity ratings for negative and basic emotions further demonstrate that valence and basicness are strongly correlated and that these emotions play a fundamental role in daily life.

In sum, this study investigated intensity ratings from a theoretical perspective. Our participants were directly asked to rate the level of intensity rather than rate the dimensions of arousal, valence, and basicness. Therefore, participants' ratings of intensity are based on their understanding of the concept of intensity, independent of the notions of arousal, valence, and basicness. These three dimensions, reduced to theory‐based binary characteristics rather than empirically measured gradients, explained intensity ratings to different extents. Arousal and Basicness are more strongly related to intensity than to Valence, corroborating previous findings that arousal and intensity are positively correlated (Laukka et al. 2005). Although four dimensions (arousal, valence, potency, and intensity) have been reported in many studies (Laukka et al. 2005; Smith and Ellsworth 1985), our results demonstrate that basicness is another important dimension of emotions. Additionally, we observed that although intensity ratings were affected by arousal, valence, and basicness, they varied across the eight emotions. Particularly, Anger and Joy were consistently rated as more intense than other emotions, both across all responses and in the subset of correct responses, highlighting their pivotal role in human emotional experience.

Also, we speculated that intensity ratings were higher for correct than for incorrect identifications of the emotion type, with the size of these differences varying across the eight emotions (see Figure 2). In the literature, there are inconsistent results regarding the impact of intensity on recognition accuracy. Some studies have demonstrated that emotions with higher intensity are better recognized than those with lower intensity (Bänziger et al. 2012; Hess et al. 1997; Juslin and Laukka 2001; Livingstone and Russo 2018; Wingenbach et al. 2016). As emotions become more intense, they are more pronounced, making them easier to identify. On the other hand, emotions with peak intensity interfere with recognition accuracy and with the rating of valence (Holz et al. 2021). Our data revealed a positive correlation between intensity and recognition accuracy, as the intensity rating was higher for correct than for incorrect ones (see Appendix C), which is consistent with the notion that emotions with stronger acoustic cues are more prominent and easier to recognize (Bachorowski and Owren 2003; Scherer 1986). However, due to the lack of peak emotions, we were unable to examine the (negative) effect of peak intensity on emotion recognition in the current study.

To assess whether the models had sufficient statistical power to test the in‐group hypothesis, we performed post hoc analyses using the *simr* package in R to investigate the main effect of speaker language. The predicted power (100 simulations) was 0.95 (95% CI [0.933, 0.962]) to identify in‐group bias in Dutch listeners across the entire dataset, and 0.76 (95% CI [0.729, 0.783]) in the subset of correct responses only. The predicted power was above or close to the threshold of 0.80, indicating sufficient power to identify an in‐group bias. For Korean listeners, the estimated power was 0.15 (95% CI [0.127, 0.173]) to test an in‐group bias in the entire dataset, and was 0.37 (95% CI [0.339, 0.400]) to identify an in‐group bias in the subset (correct responses only), suggesting that we might have found an in‐group bias with a much larger sample of participants. This approach, however, is not the right one for evaluating the in‐group hypothesis. Figure 2 shows that the speaker's language lines keep crossing each other. One line is not systematically higher than the other one, and the differences between emotions are stronger than the differences between speaker languages. This pattern points out that the interaction between emotions and language outweighs any consistent in‐group bias. This can also be inferred from the statistical information in Models 1–4 in Appendix A. The interaction effects between speaker language and emotion are stronger than the main effect of speaker language. The main effects of emotion are stronger than those of the speaker's language. In addition, the number of participants in previous studies on intensity in acoustic cues justifies the sample size of our study. For example, Juslin and Laukka (2001) tested 15 participants and found that listeners decoded portrayals with strong intensity better than those with weak intensity. Recently, Morningstar et al. (2021) tested 190 listeners to examine the impact of intensity on the recognition of vocal socioemotional expressions. Compared with previous studies, the total number of participants in our study (Dutch: 31; Korean: 24) falls within the typical range for vocal studies.

As a final note, the order of stimulus presentation constitutes a limitation of this study. In our design, the Korean recordings of 128 portrayals were presented before the Dutch recordings, and the stimuli for each participant were randomized. The effect of the sequential order on intensity ratings was minimal, as the intensity ratings of Korean and Dutch recordings did not change systematically over time. As shown in Figure S4, the mean intensity ratings across 256 stimuli, plotted as a time series, display a shallow U‐shaped trend, with a slight decrease in the first half and a slight increase in the second half. The variation is small given the wide range of intensity ratings (the blue and red points). Importantly, there is no clear discontinuity between the Korean and the Dutch parts.

## 5 Conclusion

Intensity, as a fundamental dimension of emotions, is an intriguing topic in affective sciences (Juslin and Laukka 2001; Kommattam et al. 2019; Laukka et al. 2005; Smith and Ellsworth 1985). However, theoretical and empirical studies on intensity, particularly in the vocal domain, remain sparse (Baum and Nowicki 1998). To fill this gap, we investigated cross‐cultural perception of intensity of vocal emotions by comparing ratings from Dutch and Korean listeners on the full set of stimuli in the Demo (Dutch speakers) and Koremo (Korean speakers) corpora. To our knowledge, this is the first study to investigate intensity ratings of vocal emotions using both discrete and dimensional approaches.

The first aim was to examine the hypothesized in‐group bias in intensity ratings (Ekman et al. 1987; Kommattam et al. 2019). However, our results did not convincingly support this hypothesis. Instead of in‐group bias, listeners rated particular emotions as more intense, even though they were not produced in their native language. Therefore, intensity ratings are more dependent on emotions than on the language or culture shared by both listeners and speakers. Although there were discrepancies in intensity ratings between native and non‐native listeners, the rating patterns remained basically the same. Additionally, intensity ratings are generally higher for the subset of correctly identified emotions than for the total undifferentiated response set, but the correctness of the response does not substantially interact with other factors.

The second aim was to examine the effect of arousal, valence, and basicness on intensity ratings. Arousal, valence, and intensity are three of the four fundamental dimensions of emotions (Larsen and Diener 1987; Smith and Ellsworth 1985). The fourth dimension, potency, was not included in the study. Although basicness is not classified as one of the fundamental dimensions, it plays a pivotal role in emotions (Ekman and Cordaro 2011). However, the extent to which intensity correlates with other dimensions was limited. The results reveal that intensity ratings were affected by arousal, valence, and basicness, such that intensity ratings were higher for high‐arousal than low‐arousal, higher for negative than positive, and higher for basic than non‐basic emotions. However, none of these dimensions provides a sharp dichotomy of the eight emotions in terms of intensity since intensity ratings for certain emotions cannot be reliably predicted from the general patterns.

Our results partially replicate earlier findings and provide new insights into intensity ratings of vocal emotions. Further studies are needed to examine additional emotional characteristics (e.g., potency) and their determinants of perceived emotional intensity.

## Author Contributions

Conceptualization, Methodology: Yachan Liang. Data analysis: Yachan Liang, Roeland van Hout, Vincent J. van Heuven. Visualization: Yachan Liang, Roeland van Hout, Vincent J. van Heuven. Writing – original draft: Yachan Liang. Writing – review and editing: Yachan Liang, Roeland van Hout, Vincent J. van Heuven.

## Funding

The authors have nothing to report.

## Ethics Statement

Since the data and corpus used in this study were collected by external sources and not directly gathered by the researchers, ethical approval is unnecessary.

## Conflicts of Interest

The authors declare no conflicts of interest.

## Supporting information


**Figure S1:** Means score of the intensity ratings for high‐arousal and low‐arousal emotions by Dutch and Korean listeners (A) across all responses (marginal *R* ^2^: 0.098, conditional *R* ^2^: 0.235), (B) across correct responses (marginal *R* ^2^: 0.141, conditional *R* ^2^: 0.234), and their confidence intervals (2SE).
**Figure S2:** Means score of the intensity ratings for negative and positive emotions by Dutch and Korean listeners (A) across all responses (marginal *R* ^2^: 0.028, conditional *R* ^2^: 0.084), (B) across correct responses (marginal *R* ^2^: 0.011, conditional *R* ^2^: 0.087), and their confidence intervals (2SE).
**Figure S3:** Mean intensity ratings for basic and non‐basic emotions by Dutch and Korean listeners (A) across all responses (marginal *R* ^2^: 0.121, conditional *R* ^2^: 0.258), (B) across correct responses (marginal *R* ^2^: 0.087, conditional *R* ^2^: 0.163), and their confidence intervals (2SE).
**Figure S4:** Mean intensity ratings for all data, with a smooth LOESS line to show the pattern over time (from stimulus 1 to stimulus 256).

## Data Availability

Broersma, M., Goudbeek, M., Choi, J., Konopka, A. (2025). Demo/Koremo corpus for Dutch and Korean emotional speech (Version 1) [Data set]. Radboud University. https://doi.org/10.34973/5kg3‐9852.

## Endnotes

^1^
The Korean speakers used slightly different vowel sounds “/a/” and “/o/” as substitutes for Dutch “/ɑ/” and “/ɔ/”, respectively.

## Appendix A Summary of LME Models 1 Through 10. Significant *p*‐Values (*p* ≤ 0.05) in Bold Face

**Table:**

Model 1: Linear mixed effects model for Dutch listeners, all responses	
	*Variance*
Random effects	
Listener (intercept)	0.09
Speaker language	0.04
Speaker (intercept)	0.09
Residual	0.92

	*β*	SE	*t*	*p*
Fixed effects				
Intercept	2.82	0.10	28.98	**< 0.001**
Speaker language	−0.64	0.16	−3.91	**< 0.001**
Emotion (joy)	−0.20	0.04	−4.64	**< 0.001**
Emotion (pride)	−0.89	0.04	−20.61	**< 0.001**
Emotion (fear)	−0.27	0.04	−6.28	**< 0.001**
Emotion (tenderness)	−1.23	0.04	−28.54	**< 0.001**
Emotion (relief)	−0.96	0.04	−22.27	**< 0.001**
Emotion (sadness)	−0.69	0.04	−15.99	**< 0.001**
Emotion (irritation)	−1.07	0.04	−24.77	**< 0.001**
Speaker language × Emotion (joy)	0.10	0.09	1.22	0.223
Speaker language × Emotion (pride)	0.85	0.09	9.84	**< 0.001**
Speaker language × Emotion (fear)	0.50	0.09	5.76	**< 0.001**
Speaker language × Emotion (tenderness)	0.47	0.09	5.46	**< 0.001**
Speaker language × Emotion (relief)	0.53	0.09	6.21	**< 0.001**
Speaker language × Emotion (sadness)	1.00	0.09	11.64	**< 0.001**
Speaker language × Emotion (irritation)	0.52	0.09	6.04	**< 0.001**

Model 2: Linear mixed effects model for Korean listeners, all responses	
	Variance
Random effects	
Listener (Intercept)	0.15
Speaker Language	0.04
Speaker (Intercept)	0.09
Residual	0.93

	*β*	SE	*t*	*p*
Fixed effects				
Intercept	3.14	0.11	27.42	**< 0.001**
Speaker language	−0.15	0.17	−0.90	0.380
Emotion (joy)	−0.40	0.05	−8.21	**< 0.001**
Emotion (pride)	−0.99	0.05	−20.16	**< 0.001**
Emotion (fear)	−0.48	0.05	−9.82	**< 0.001**
Emotion (tenderness)	−1.23	0.05	−25.02	**< 0.001**
Emotion (relief)	−1.24	0.05	−25.04	**< 0.001**
Emotion (sadness)	−0.87	0.05	−17.66	**< 0.001**
Emotion (irritation)	−1.34	0.05	−27.21	**< 0.001**
Speaker language × Emotion (joy)	−0.38	0.10	−3.88	**< 0.001**
Speaker language × Emotion (pride)	0.55	0.10	5.54	**< 0.001**
Speaker language × Emotion (fear)	0.01	0.10	0.11	0.916
Speaker language × Emotion (tenderness)	0.37	0.10	3.80	**< 0.001**
Speaker language × Emotion (relief)	0.11	0.10	1.08	0.279
Speaker language × Emotion (sadness)	0.45	0.10	4.57	**< 0.001**
Speaker language × Emotion (irritation)	0.40	0.10	4.04	**< 0.001**

Model 3: Linear mixed effects model analyses for Dutch listeners, correct responses	
	Variance
Random effects	
Listener (Intercept)	0.10
Speaker Language	0.04
Speaker (Intercept)	0.08
Residual	0.67

	*β*	SE	*t*	*p*
Fixed effects				
Intercept	3.19	0.10	31.84	**< < 0.001**
Speaker language	−0.48	0.17	−2.84	**< 0.010**
Emotion (joy)	−0.32	0.06	−5.09	**< 0.001**
Emotion (pride)	−0.88	0.09	−10.21	**< 0.001**
Emotion (fear)	−0.44	0.05	−8.05	**< 0.001**
Emotion (tenderness)	−0.96	0.07	−14.60	**< 0.001**
Emotion (relief)	−0.97	0.06	−17.50	**< 0.001**
Emotion (sadness)	−0.83	0.05	−17.01	**< 0.001**
Emotion (irritation)	−1.12	0.05	−20.85	**< 0.001**
Speaker language × Emotion (joy)	−0.09	0.13	−0.70	0.482
Speaker language × Emotion (pride)	0.74	0.17	4.27	**< 0.001**
Speaker language × Emotion (fear)	0.40	0.11	3.69	**< 0.001**
Speaker language × Emotion (tenderness)	0.50	0.13	3.82	**< 0.001**
Speaker language × Emotion (relief)	0.34	0.11	3.08	**< 0.010**
Speaker language × Emotion (sadness)	0.76	0.10	7.84	**< 0.001**
Speaker language × Emotion (irritation)	0.50	0.11	4.65	**< 0.001**

Model 4: Linear mixed effects model analyses for Korean listeners, correct responses	
	Variance
Random effects	
Listener (intercept)	0.13
Speaker language	0.08
Speaker (intercept)	0.06
Residual	0.69

	*β*	SE	*t*	*p*
Fixed effects				
Intercept	3.28	0.10	31.36	**< 0.001**
Speaker language	−0.26	0.16	−1.64	0.109
Emotion (joy)	−0.32	0.08	−3.91	**< 0.001**
Emotion (pride)	−0.63	0.08	−7.79	**< 0.001**
Emotion (fear)	−0.55	0.07	−7.86	**< 0.001**
Emotion (tenderness)	−0.64	0.08	−7.65	**< 0.001**
Emotion (relief)	−1.16	0.07	−17.07	**< 0.001**
Emotion (sadness)	−0.84	0.06	−14.95	**< 0.001**
Emotion (irritation)	−1.25	0.07	−17.68	**< 0.001**
Speaker language × Emotion (joy)	−0.43	0.16	−2.61	**< 0.010**
Speaker language × Emotion (pride)	0.90	0.16	5.62	**< 0.001**
Speaker language × Emotion (fear)	0.21	0.14	1.50	0.135
Speaker language × Emotion (tenderness)	0.49	0.17	2.94	**< 0.010**
Speaker language × Emotion (relief)	0.32	0.14	2.39	**< 0.050**
Speaker language × Emotion (sadness)	0.61	0.11	5.36	**< 0.001**
Speaker language × Emotion (irritation)	0.55	0.14	3.94	**< 0.001**

Model 5: Linear mixed effects model analyses for Arousal, all responses	
	Variance
Random effects	
Listener (intercept)	0.10
Arousal	0.04
Listener (intercept)	0.02
Speaker language	0.04
Speaker (intercept)	0.04
Arousal	0.07
Speaker (intercept)	0.06
Listener language	0.00
Residual	0.99

	*β*	SE	*t*	*p*
Fixed effects				
Intercept	2.24	0.09	23.89	**< 0.001**
Speaker language	−0.05	0.16	−0.34	0.743
Listener language	0.17	0.10	1.72	0.091
Arousal	−0.67	0.08	−8.95	**< 0.001**
Speaker language × Listener language	0.18	0.06	2.76	**< 0.010**
Speaker language × Arousal	0.28	0.14	2.01	0.064
Listener language × Arousal	−0.05	0.07	−0.80	0.425
Speaker language × Listener language × Arousal	0.02	0.07	0.30	0.766

Model 6: Linear mixed effects model analyses for Arousal, correct responses	
	Variance
Random effects	
Listener (intercept)	0.03
Arousal	0.04
Listener (intercept)	0.07
Speaker language	0.05
Speaker (intercept)	0.01
Arousal	0.09
Speaker (intercept)	0.05
Listener language	0.01
Residual	0.71

	*β*	SE	*t*	*p*
Fixed effects				
Intercept	2.60	0.08	32.86	**< 0.001**
Speaker language	−0.05	0.14	−0.39	0.700
Listener language	0.09	0.10	0.98	0.330
Arousal	−0.68	0.08	−8.36	**< 0.001**
Speaker language × Listener language	0.10	0.10	1.07	0.293
Speaker language × Arousal	0.44	0.15	2.85	**< 0.050**
Listener language × Arousal	−0.01	0.07	−0.16	0.872
Speaker language × Listener language × Arousal	0.05	0.09	0.53	0.595

Model 7: Linear mixed effects model analyses for Valence, all responses	
	Variance
Random effects	
Listener (intercept)	0.04
Listener (intercept)	0.08
Speaker language	0.04
Speaker (intercept)	0.01
Valence	0.08
Speaker (intercept)	0.08
Listener language	0.00
Residual	0.10

	*β*	SE	*t*	*p*
Fixed effects				
Intercept	2.24	0.09	25.00	**< 0.001**
Speaker language	−0.05	0.16	−0.35	0.729
Listener language	0.17	0.09	1.78	0.080
Valence	−0.30	0.07	−4.27	**< 0.001**
Speaker language × Listener language	0.18	0.06	2.80	**< 0.010**
Speaker language × Valence	−0.03	0.14	−0.24	0.811
Listener language × Valence	0.02	0.04	0.54	0.588
Speaker language × Listener language × Valence	−0.04	0.07	−0.53	0.596

Model 8: Linear mixed effects model analyses for Valence, correct responses	
	Variance
Random effects	
Listener (intercept)	0.05
Valence	0.03
Listener (intercept)	0.06
Speaker language	0.06
Speaker (intercept)	0.00
Valence	0.07
Speaker (intercept)	0.06
Listener language	0.01
Residual	0.84

	*β*	SE	*t*	*p*
Fixed effects				
Intercept	2.52	0.08	32.19	**< 0.001**
Speaker language	−0.06	0.13	−0.43	0.674
Listener language	0.09	0.10	0.95	0.348
Valence	−0.17	0.08	−2.23	**< 0.050**
Speaker language × Listener language	0.13	0.10	1.33	0.192
Speaker language × Valence	0.02	0.14	0.14	0.892
Listener language × Valence	0.07	0.07	1.06	0.294
Speaker language × Listener language × Valence	0.33	0.11	3.07	**< 0.010**

Model 9: Linear mixed effects model analyses for Basicness, all responses	
	Variance
Random effects	
Listener (intercept)	0.09
Basicness	0.04
Listener (intercept)	0.03
Speaker language	0.04
Speaker (intercept)	0.05
Basicness	0.11
Speaker (intercept)	0.04
Listener language	0.00
Residual	0.96

	*β*	SE	*t*	*p*
Fixed effects				
Intercept	2.24	0.09	25.12	**< 0.001**
Speaker language	−0.05	0.15	−0.36	0.727
Listener language	0.17	0.10	1.77	0.082
Basicness	−0.75	0.09	−8.68	**< 0.001**
Speaker language × Listener language	0.18	0.07	2.74	**< 0.010**
Speaker language × Basicness	0.26	0.17	1.60	0.132
Listener language × Basicness	−0.02	0.06	−0.26	0.789
Speaker language × Listener language × Basicness	0.15	0.07	2.18	**< 0.050**

Model 10: Linear mixed effects model analyses for Basicness, correct responses	
	Variance
Random effects	
Listener (intercept)	0.01
Basicness	0.02
Listener (intercept)	0.10
Speaker language	0.05
Speaker (intercept)	0.02
Basicness	0.15
Speaker (intercept)	0.04
Listener language	0.01
Residual	0.75

	*β*	SE	*t*	*p*
Fixed effects				
Intercept	2.48	0.08	31.22	**< 0.001**
Speaker Language	−0.03	0.13	−0.22	0.826
Listener Language	0.08	0.10	0.88	0.385
Basicness	−0.54	0.10	−5.36	**< 0.001**
Speaker Language × Listener Language	0.19	0.09	2.09	**< 0.050**
Speaker Language × Basicness	0.22	0.20	1.09	0.295
Listener Language × Basicness	0.11	0.06	1.80	0.078
Speaker Language × Listener Language × Basicness	0.23	0.10	2.34	**< 0.050**

## Appendix B Intercepts for Arousal, Valence, and Basicness for Eight Emotions, for All Responses, and for Correctly Identified Emotions Only

**Table:**

Emotion	Arousal		Valence		Basicness	
	All	Correct	All	Correct	All	Correct
Anger	0.39	0.39	0.58	0.66	0.35	0.45
Joy	0.11	0.09	0.59	0.46	0.07	0.11
Fear	0.03	−0.10	0.22	0.11	−0.01	−0.10
Sadness	0.30	0.13	−0.19	−0.24	−0.41	−0.46
Pride	−0.53	−0.38	−0.05	−0.03	0.17	0.18
Relief	−0.01	−0.08	−0.20	−0.33	0.03	−0.07
Irritation	−0.12	−0.19	−0.61	−0.54	−0.08	−0.23
Tenderness	−0.16	0.15	−0.35	−0.11	−0.12	0.12

## Appendix C Recognition Accuracy (%) and Intensity Ratings (0–4) by Dutch and Korean Listeners, for Dutch and Korean Speakers

**Table:**

Emotion	Dutch listeners				Korean listeners			
	Dutch speakers		Korean speakers		Dutch speakers		Korean speakers	
	Accuracy	Intensity	Accuracy	Intensity	Accuracy	Intensity	Accuracy	Intensity
Anger	62.28	3.14	37.50	2.50	64.95	3.22	33.42	3.07
Joy	42.46	2.89	22.41	2.35	20.92	3.01	21.20	2.47
Pride	20.91	1.83	8.19	2.03	20.11	1.95	24.73	2.35
Fear	40.95	2.62	53.88	2.47	26.09	2.73	51.09	2.59
Tenderness	29.74	1.67	23.71	1.50	14.40	1.80	34.24	2.02
Relief	48.28	1.91	41.59	1.80	36.14	1.93	39.40	1.89
Sadness	80.82	1.95	73.49	2.31	82.61	2.12	81.25	2.42
Irritation	53.45	1.81	44.40	1.69	64.95	3.22	33.42	3.07
