# Supplementary material for: Interpreting the Intensity of Vocal Emotions Across Cultures
Source: Scand J Psychol. 2026 Feb 19;67(4):983–1003. doi: 10.1111/sjop.70081 (PMC13352553; doi:10.1111/sjop.70081)
Supplement: Supplementary file 1 — Figure S1: Means score of the intensity ratings for high‐arousal and low‐arousal emotions by Dutch and Korean listeners (A) across all responses (marginal R 2: 0.098, conditional R 2: 0.235), (B) across correct responses (marginal R 2: 0.141, conditional R 2: 0.234), and their confidence intervals (2SE). Figure S2: Means score of the intensity ratings for negative and positive emotions by Dutch and Korean listeners (A) across all responses (marginal R 2: 0.028, conditional R 2: 0.084), (B) across correct responses (marginal R 2: 0.011, conditional R 2: 0.087), and their confidence intervals (2SE). Figure S3: Mean intensity ratings for basic and non‐basic emotions by Dutch and Korean listeners (A) across all responses (marginal R 2: 0.121, conditional R 2: 0.258), (B) across correct responses (marginal R 2: 0.087, conditional R 2: 0.163), and their confidence intervals (2SE). Figure S4: Mean intensity ratings for all data, with a smooth LOESS line to show the pattern over time (from stimulus 1 to stimulus 256). [file SJOP-67-983-s001.docx]

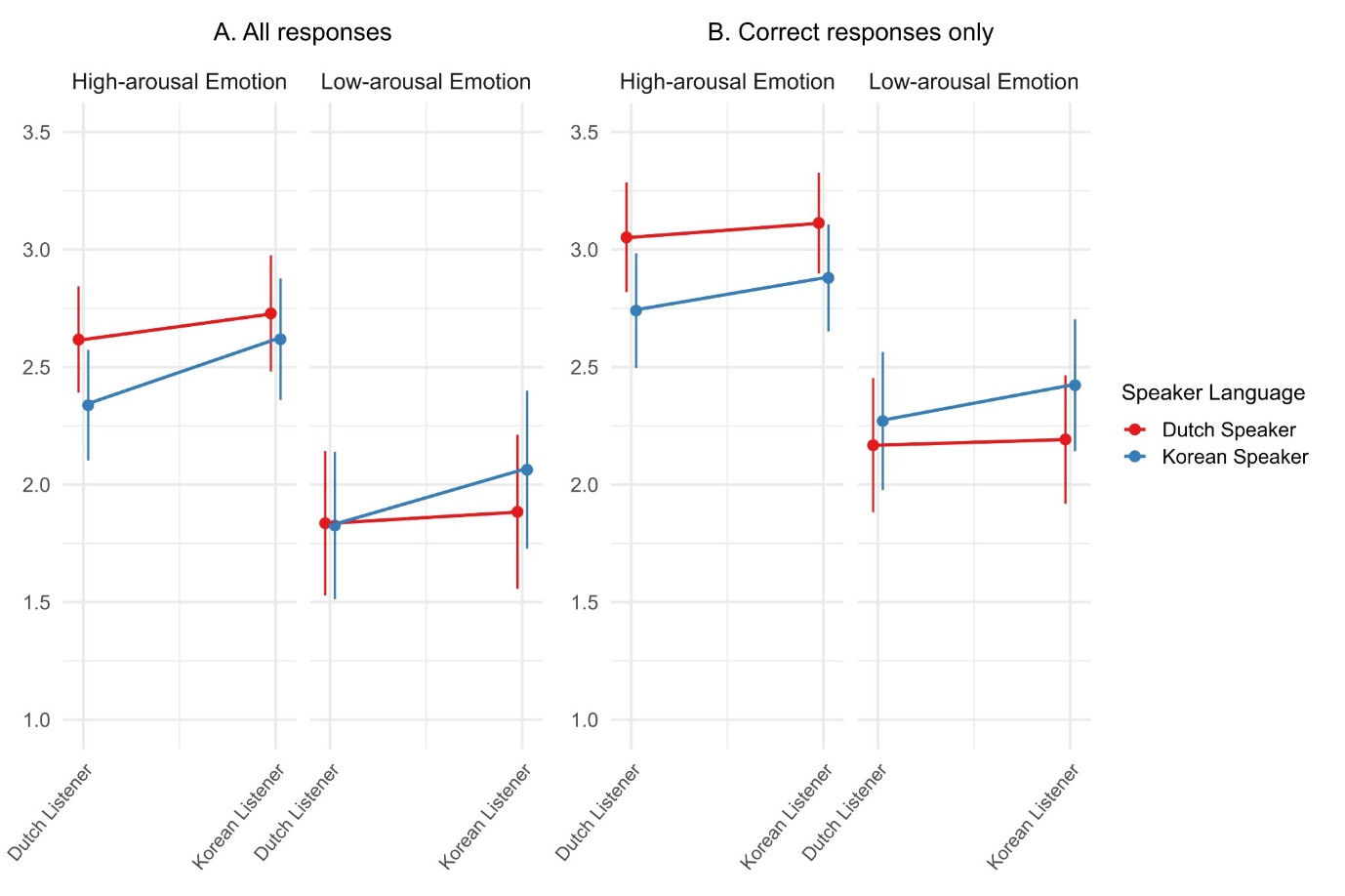
**Supplementary materials**

**Figure S1**. Means score of the intensity ratings for high-arousal and low-arousal emotions by Dutch and Korean listeners (A) across all responses (marginal R^2^: 0.098, conditional R^2^: 0.235), (B) across correct responses (marginal R^2^: 0.141, conditional R^2^: 0.234), and their confidence intervals (2SE).


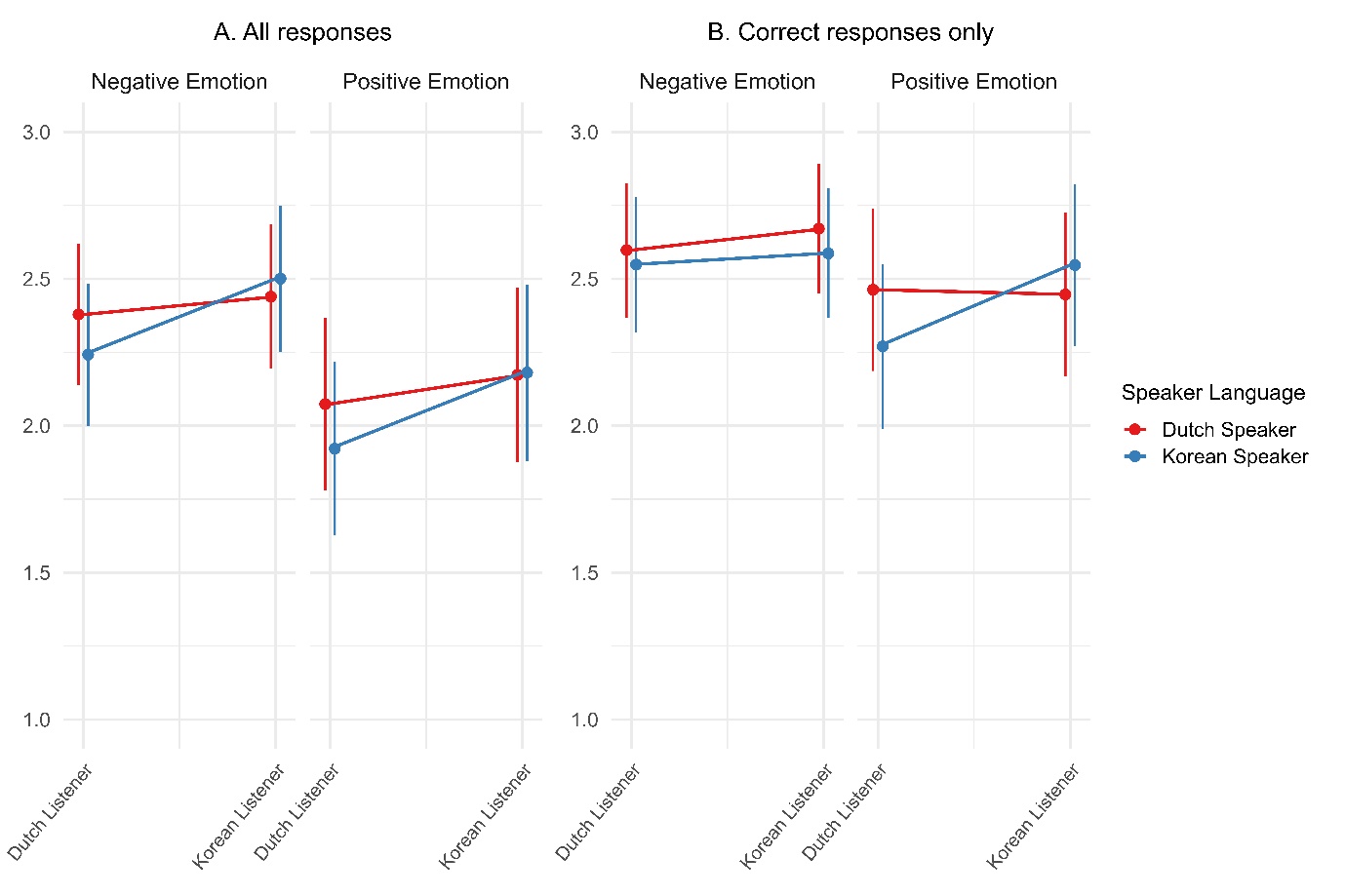


**Figure S2**. Means score of the intensity ratings for negative and positive emotions by Dutch and Korean listeners (A) across all responses (marginal R^2^: 0.028, conditional R^2^: 0.084), (B) across correct responses (marginal R^2^: 0.011, conditional R^2^: 0.087), and their confidence intervals (2SE).


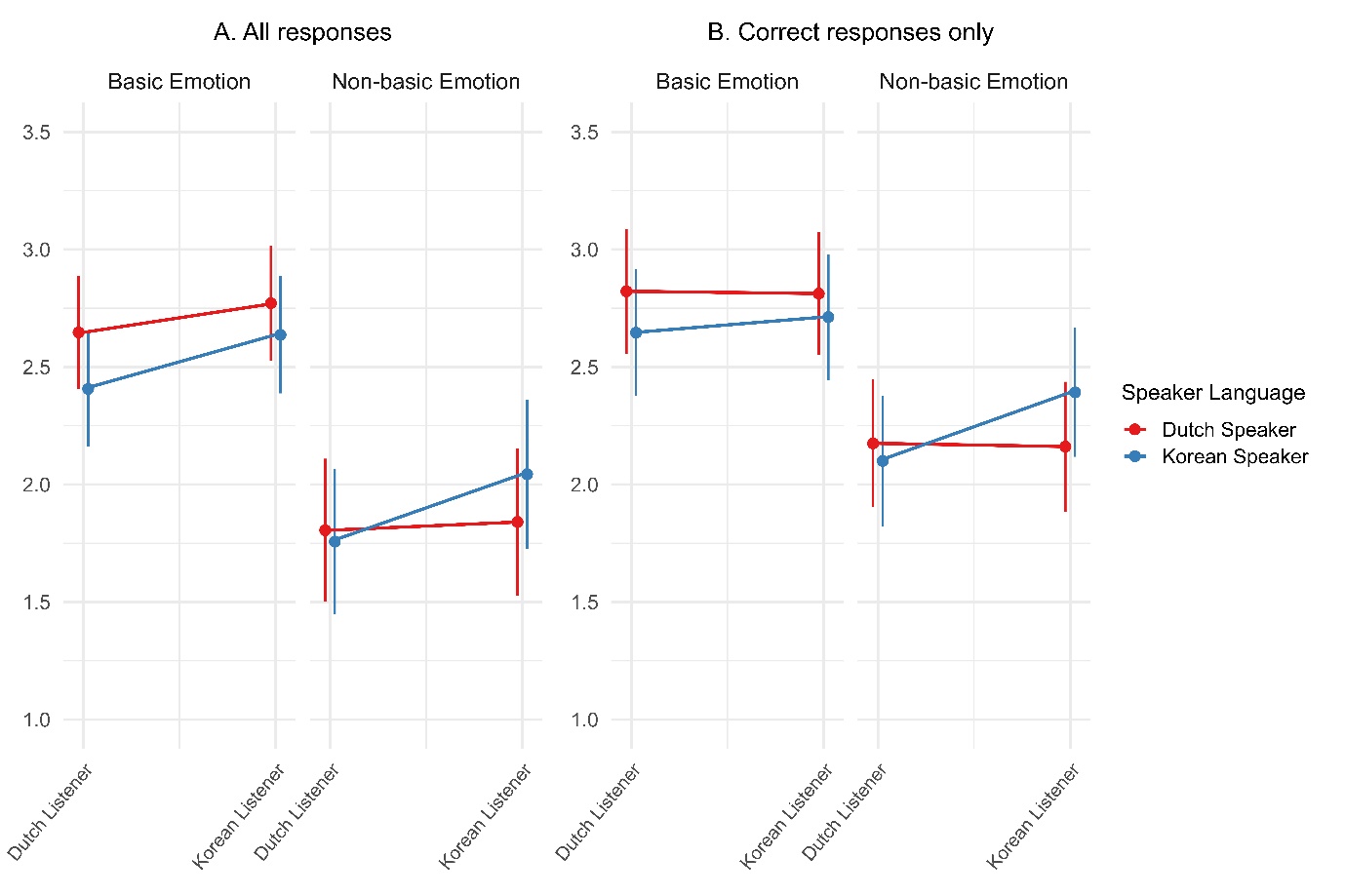


**Figure S3**. Mean intensity ratings for basic and non-basic emotions by Dutch and Korean listeners (A) across all responses (marginal R^2^: 0.121, conditional R^2^: 0.258), (B) across correct responses (marginal R^2^: 0.087, conditional R^2^: 0.163), and their confidence intervals (2SE).


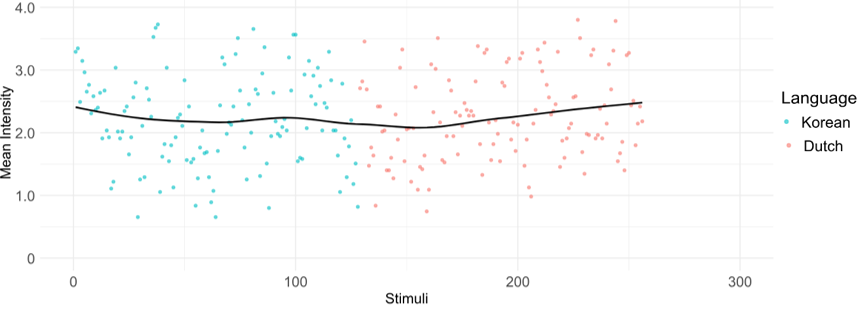


**Figure S4**. Mean intensity ratings for all data, with a smooth LOESS line to show the pattern over time (from stimulus 1 to stimulus 256).
